# Glycerophosphodiester phosphodiesterase 1 (GDE1) acts as a potential tumor suppressor and is a novel therapeutic target for non-mucin-producing colon adenocarcinoma

**DOI:** 10.7717/peerj.8421

**Published:** 2020-02-11

**Authors:** Qiu Shen, Chao Lu, Hua Yang, Ming-Xia Ge, Wang-Xiao Xia, Qing-Peng Kong, Gong-Hua Li, Yan-Hong Gu

**Affiliations:** 1 Department of Oncology, The First Affiliated Hospital of Nanjing Medical University, Nanjing, Jiangsu, China; 2 State Key Laboratory of Genetic Resources and Evolution/Key Laboratory of Healthy Aging Research of Yunnan Province, Kunming Institute of Zoology, Chinese Academy of Sciences, Kunming, Yunnan, China; 3 Department of Oncology, Jiangyin People’s Hospital, Wuxi, Jiangsu, China; 4 The Third People’s Hospital of Yunnan Province, Kunming, Yunnan, China

**Keywords:** Colon adenocarcinoma, Non-mucin-producing colon adenocarcinoma, Metabolism, Histological subtype, Prognosis, Weighted gene co-expression network analysis (WGCNA), Glycerophosphodiester phosphodiesterase 1

## Abstract

Colon adenocarcinoma (COAD) represents a major public health issue due to its high incidence and mortality. As different histological subtypes of COAD are related to various survival outcomes and different therapies, finding specific targets and treatments for different subtypes is one of the major demands of individual disease therapy. Interestingly, as these different subtypes show distinct metabolic profiles, it may be possible to find specific targets related to histological typing by targeting COAD metabolism. In this study, the differential expression patterns of metabolism-related genes between COAD (*n* = 289) and adjacent normal tissue (*n* = 41) were analyzed by one-way ANOVA. We then used weighted gene co-expression network analysis (WGCNA) to further identify metabolism-related gene connections. To determine the critical genes related to COAD metabolism, we obtained 2,114 significantly differentially expressed genes (DEGs) and 12 modules. Among them, we found the hub module to be significantly associated with histological typing, including non-mucin-producing colon adenocarcinoma and mucin-producing colon adenocarcinoma. Combining survival analysis, we identified glycerophosphodiester phosphodiesterase 1 (GDE1) as the most significant gene associated with histological typing and prognosis. This gene displayed significantly lower expression in COAD compared with normal tissues and was significantly correlated with the prognosis of non-mucin-producing colon adenocarcinoma (*p* = 0.0017). Taken together, our study showed that GDE1 exhibits considerable potential as a novel therapeutic target for non-mucin-producing colon adenocarcinoma.

## Introduction

Colorectal cancer is a common type of digestive cancer worldwide. Its incidence has increased rapidly in recent years and seriously impacts human health. In 2018, over 1.8 million new cases and 881,000 deaths were estimated to have occurred, accounting for about 1 in 10 cancer cases and deaths worldwide (Bray et al., 2018). Among cancer-related diseases, colorectal cancer ranks third in terms of incidence and second in terms of mortality (Bray et al., 2018). With changes in people’s lifestyles and dietary habits, China has experienced an upward trend in both the incidence and mortality of colorectal cancer in the last decade, which now rank fourth and fifth among cancers, respectively (Ministry of Health of the People’s Republic of China, 2016). Unlike other cancers, no single risk factor accounts for most cases of colorectal cancer (Brenner, Kloor & Pox, 2014). Dietary intake, nutritional status, physical activity and other changes have shown to be associated with pathogenesis and poor outcomes of colorectal cancer (Thanikachalam & Khan, 2019). Widely epidemiological and observational evidence showed the risk of colorectal cancer is strictly related to lifestyle, especially to diet and physical activity (World Cancer Research Fund/American Institute for Cancer Research, 2007). In analytical case-control and cohort studies, risk is directly associated with the consumption of red and processed meat (Roncucci & Mariani, 2015).

The most common colorectal cancer is colon adenocarcinoma (COAD), which accounts for 66.1% of the disease (Ji et al., 2017). Overall COAD survival has not substantially improved over the past few decades (Liu, Chen & Xu, 2018) and its prognosis remains poor due to the delay in diagnosis, advanced stage of disease, and lack of histology-specific treatment (Al-Tonbary, Darwish & El-Hussein, 2013). Therefore, identification of novel biomarkers for early diagnosis and development of histology-specific targets is one of the foremost challenges for individual treatment of COAD.

There is a strong and multifaceted connection between cell metabolism and cancer (Vander Heiden, Cantley & Thompson, 2009). Altered cellular metabolism can meet cancer cell anabolic demands that result from untethered cellular growth and aberrant differentiation. Metabolic reprogramming is widely observed during cancer development to confer cancer cells the ability to survive and proliferate, even under stressful conditions, such as limited nutrients (Li & Zhang, 2016). Energy metabolism reprogramming, which fuels fast cell growth and proliferation by adjustments in energy metabolism, is considered an emerging hallmark of cancer (Hanahan & Weinberg, 2011). Thus, new strategies for treating various malignancies by targeting cancer metabolism are gaining increasing attention.

In the current study, we aimed to identify potential targets involved in COAD metabolism by performing comprehensive transcriptome-wide analysis of COAD gene expression patterns. We systematically analyzed clusters of genes with similar expression patterns using weighted gene co-expression network analysis (WGCNA) and found the MEred module to be highly associated with histological typing. Further analysis of the MEred module identified glycerophosphodiester phosphodiesterase 1 (GDE1) as the most significant gene associated with COAD histological typing prognosis. Thus, GDE1 may serve as a candidate biomarker in COAD metabolism and may be a key gene for histology-specific treatment.

## Materials and Methods

### Data collection

We downloaded 289 COAD tumor samples (transcripts per million, TPM) from The Cancer Genome Atlas (TCGA, https://portal.gdc.cancer.gov/repository) and 41 peritumoral normal tissues of COAD (TPM) from the UCSC Xena website (https://xena.ucsc.edu/). The data were analyzed with a unifying pipeline, which used a CutAdapt trimming adapter, STAR for alignment, and RSEM and Kallisto as quantifiers. The COAD clinical data were downloaded from the TCGA database using the ‘cgdsr’ package (Null, Team & Null, 2009; Jacobsen, 2015).

### Differentially expressed gene (DEG) screening

We downloaded metabolism-associated genes from RECON3, the most comprehensive human metabolic network model which contains 13543 reaction, 4140 metabolites, and 3288 metabolic genes (Brunk et al., 2018), and identified 3,267 genes that corresponded to the TPM data. We removed lowly expressed genes (TPM <1) from the COAD and normal samples, and thus retained 2,747 genes. We used principal component analysis (PCA). To obtain significantly changed genes in COAD, we used the ‘aov’ function to analyze the DEGs between the COAD and normal samples. We obtained 2,114 significant DEGs in COAD with a *p* < 0.01 cutoff.

### Co-expression network construction by WGCNA

WGCNA can be used to analyze global gene expression profiling and identify highly co-expressed genes. We installed WGCNA with Bioconductor (http://bioconductor.org/biocLite.R) for co-expression analysis of metabolism-related genes in COAD. The soft threshold method was used for correlation analysis of the expression profiles. We used average linkage hierarchical clustering to group transcripts based on topological overlap dissimilarity in network connection strengths. We set the minimum gene number of each module to 30 and cutHeight parameter to 0.25 to merge similar modules.

### Identification of significant histological type modules

To incorporate external information into the co-expression network, WGCNA uses gene significance measures GS (minus log of a *p*-value). A gene significance measure indicates (the absolute value of) the correlation between individual gene in a given module and the trait. The average GS across the module genes was used to characterize the correlation between modules and clinical traits. For each module, we also define a quantitative measure of module membership MM as the correlation of the module eigengene MEs (defined as the first principal component of a given module) and the gene expression profile. The relationships between MEs and clinical traits were analyzed, and the relevant modules were identified.

### Functional and pathway enrichment analysis

We used the Database for Annotation Visualization and Integrated Discovery (DAVID) (http://david.abcc.ncifcrf.gov/) for gene functional annotation to determine biological functions. The genes in the significant histological type modules were uploaded in DAVID for Gene Ontology (GO) and Kyoto Encyclopedia of Genes and Genomes (KEGG) pathway enrichment analyses. We set a cutoff of adjusted *p* < 0.05 for significant biological processes and significant pathways.

### Survival analysis

The prognosis profiles of the genes in significant histological type modules were analyzed using the R survival package from Bioconductor (https://www.bioconductor.org). Samples were classified as two subtypes based on gene expression. Samples with larger than median gene expression levels were defined as the “high expression” subtype; all other samples were defined as the “low expression” subtype. The log-rank *p*-value and survival visualization were performed using the R “survminer” package.

## Results

### Metabolism-related DEGs in COAD

**Figure 1:**
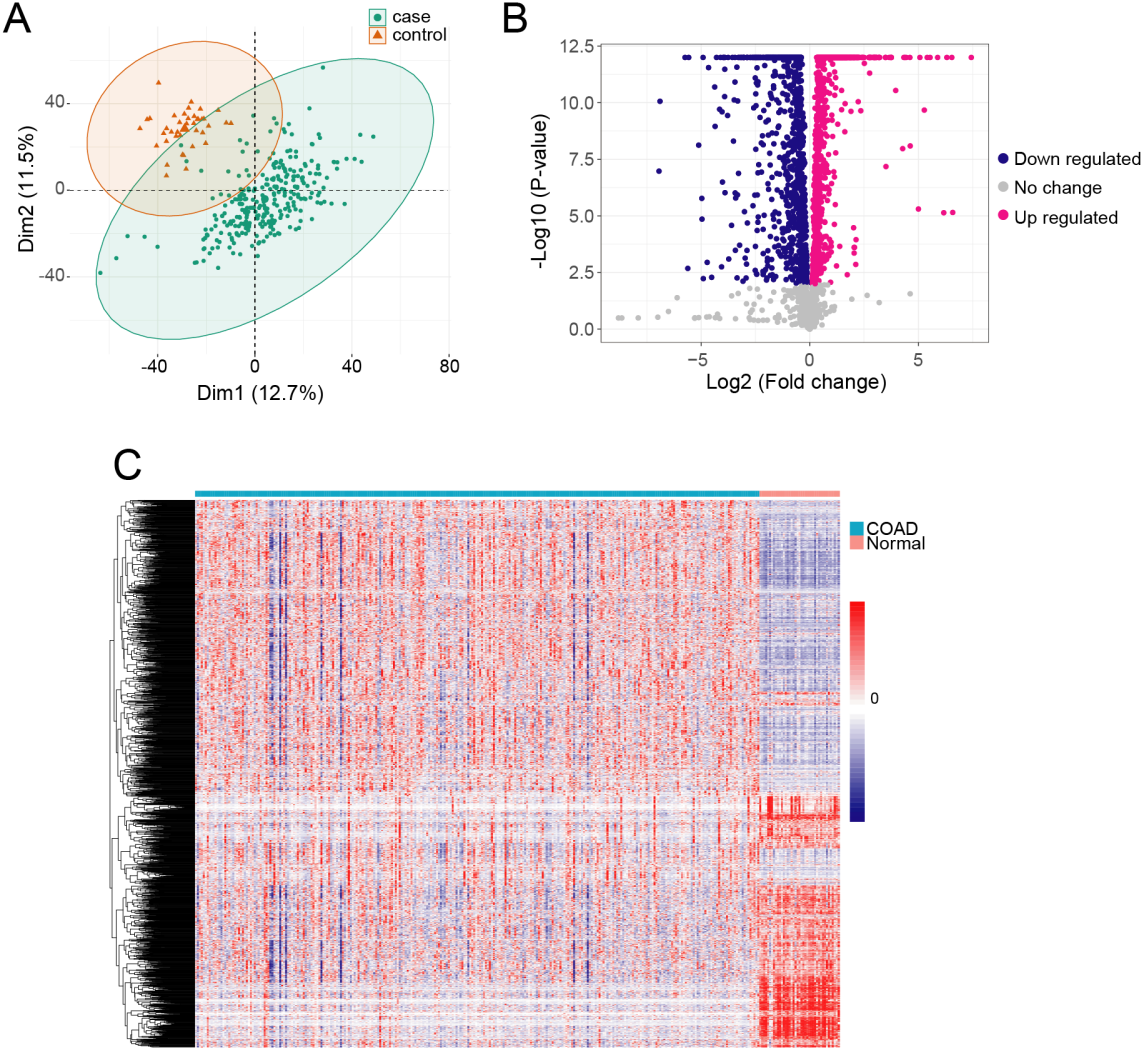
The metabolism related DEGs in COAD. (A) The PCA was performed based on gene TPM of COAD and normal samples. (B) *X*-axis and *y*-axis of the Volcano plot represent gene expression fold-changes and negative logarithm to the base 10 of *p*-values, respectively. Red nodes indicate DEGs up regulated in COAD with *p*-value of <0.01. Blue nodes indicate DEGs down regulated in COAD with *p*-value of <0.01. (C) The heatmap of metabolism related DEGs in COAD. Each column represents a sample and each row represents one gene.

We obtained gene expression TPM data from 289 COAD samples and 41 adjacent normal tissue samples, which included 60,498 genes. The QC analyzing result showed that the median and the quartile log 2 TPM value are similar between adjacent normal samples and cancer samples, indicating they are comparable (Fig. S1). We used PCA to identify the possible outlier samples, and found that all the samples are located in the confidential ellipse (Fig. 1A). We thus remained all of the samples to further analysis. To determine if metabolism-related genes influenced COAD, we first obtained 3,288 metabolism genes in RECON3, a most comprehensive human metabolic network model (Brunk et al., 2018). We removed lowly expressed genes (TPM <1) from the COAD and normal samples, and thus acquired 2,722 genes. We then determined DEGs in COAD compared with the normal tissue samples using ANOVA in R. We identified 2,114 DEGs with a cutoff of *p* < 0.01 (Fig. 1B), which included 1224 highly expressed genes and 890 lowly expressed genes (Table S1). The profiles of the 2,114 DEGs are shown with heatmap in Fig. 1C.

### Metabolism gene co-expression network constructed by WGCNA

**Figure 2:**
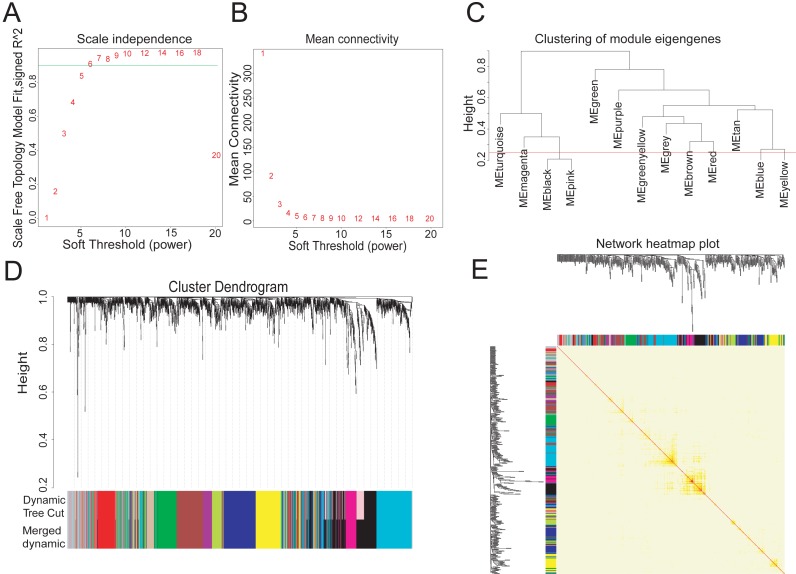
Twelve WGCNA modules of DEGs in COAD. (A–B) Soft-thresholding power analysis plot to fit the index of network topology. (C) Dendrogram of consensus module eigengenes from WGCNA analysis. The red line is the merging threshold, and groups of eigengenes below the threshold correlation need merged. (D) Hierarchical plot of co-expression clusters with corresponding color assignments. Each color represents a co-expression module by WGCNA. (E) Heatmap of the Topological Overlap Matrix (TOM) among 500 randomly selected genes from the DEG weighted co-expression network. Light and deep colors represent lower and higher overlap, respectively.

To explore the hub metabolism-related genes in COAD, we analyzed the co-expression network of the 2,114 DEGs using WGCNA. The power value is the most critical parameter that can affect the independence and average connectivity degree of co-expression modules. We screened network topology using different soft thresholding powers for later analysis (Figs. 2A, 2B). The scale-free R2 equated to 0.90 at the power value of six. Therefore, the power value of six was used to construct the co-expression network with WGCNA, which was based on the hierarchical clustering of the calculated dissimilarities. The distance between clusters was defined as the average distance between all inter-cluster pairs. With a cutHeight of 0.25 (Fig. 2C) and minimum gene number of 30, we merged the modules, with 12 then obtained (Fig. 2D). We randomly selected 500 genes in the hierarchical clustering results, with the rows and columns representing each gene in each module. The deep red color indicated high topological overlap, suggesting that the co-expressed genes had similarity at the network topology level (Fig. 2E, Fig. S2).

### Identifying genes in red module associated with histological typing

**Figure 3:**
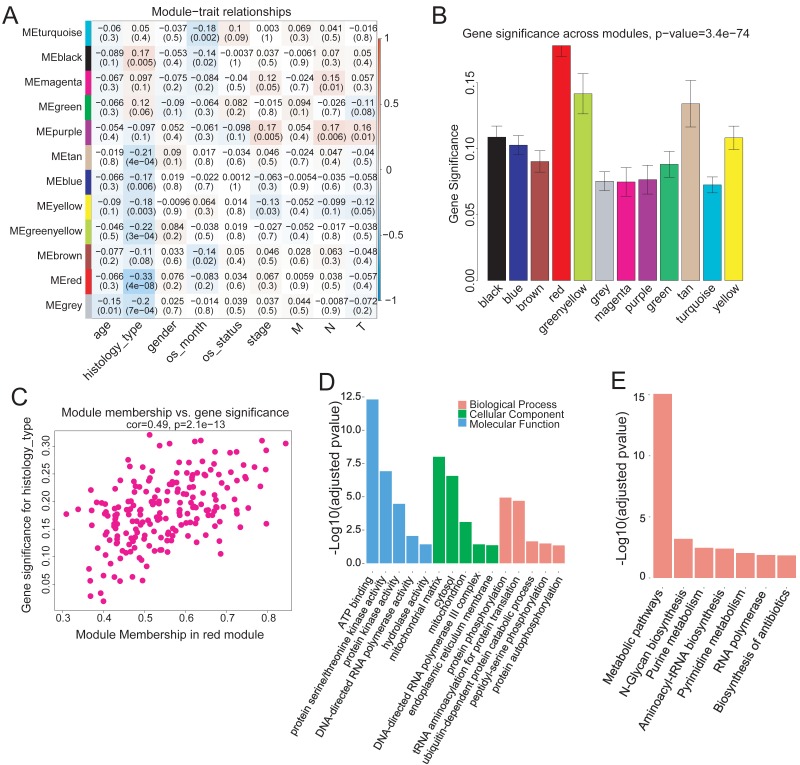
Correlation of Red module with clinical histological type. (A) Heatmap of model-trait relationship. Each cell include the the corresponding correlation and *p*-value. Each row and column corresponds to a module eigengene and a COAD clinical trait,respectively. The result indicated that the MEred module is highly correlated with the histological type with the *p*-value of 4e–8. COAD clinical traits(histology type: M-COAD,N-COAD; os_month:survival time(months); os_status:deceased or living; stage:AJCC_PATHOLOGIC_TUMOR_STAGE; M:AJCC_METASTASIS_PATHOLOGIC_PM; N:AJCC_NODES_PATHOLOGIC_PN; T: AJCC_TUMOR_PATHOLOGIC_PT_). (B) Bar plot of gene significance across the WGCNA modules.The red module shows much higher GS than any other modules (C) Correlation between MEred membership and gene significance. (D) GO and (E) KEGG enrichment analysis of 199 genes in MEred. *Y*-axis is the significance of the enrichment results with the ‘–log10 (adjusted *p*-value)’.

To investigate the modules associated with clinical traits, we analyzed the module trait relationship. We first convert clinical trait data into variables which expressed in quantitative form, next we quantify gene relationship to clinical traits and important modules by using the gene significance and module membership (MM) measures. The results showed that the red module was significantly but negatively correlated with COAD clinical histological typing (*r* =  − 0.33, *p* = 4*e*−08) (Fig. 3A). We also tested the relevance between each module and COAD clinical traits. The red module demonstrated much higher module significance than any other module, suggesting its stronger connection with COAD histological typing (Fig. 3B), viz., non-mucin-producing colon adenocarcinoma (N-COAD) and mucin-producing colon adenocarcinoma (M-COAD). Red module membership was also significantly correlated with gene significance (Fig. 3C). Taken together, our results indicated that the red module was significantly correlated to COAD histological typing.

The red module contained 199 genes (Table S2). To clarify the function of these genes, GO and KEGG pathway analyses were conducted using DAVID (v6.8). Genes in the red module were significantly enriched in ATP-binding (Benjamini–Hochberg method adjusted *p*-value = 4.83e−13) molecular function (Fig. 3D) and in N-Glycan biosynthesis pathways (Benjamini–Hochberg method adjusted *p*-value = 6.23e−04) (Fig. 3E).

### Significant association of GDE1 gene with histological typing and prognosis

**Figure 4:**
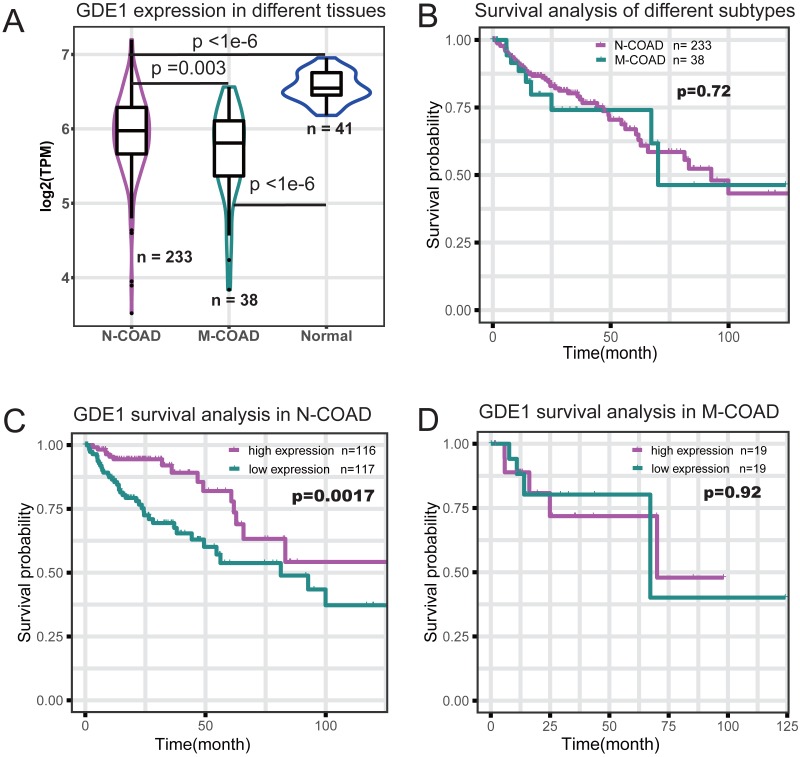
Survival profile of GDE1 in different histological typing of COAD. (A) Gene expression profile of GDE1 in normal (*n* = 41), M-COAD (*n* = 38), and N-COAD (*n* = 233). (B) Survival curve in M-COAD (*n* = 38) and N-COAD (*n* = 233). (C) Survival curves of N-COAD with high (magenta, *n* = 116) and low (dark cyan, *n* = 117) GED1 expression. (D) Survival curves of M-COAD with high (magenta, *n* = 19) and low (dark cyan, *n* = 19) GED1 expression.

To identify which genes in the red module were associated with different histological typing and prognosis of COAD, we performed survival analysis (Table 1). We found GDE1 to be the most significant prognosis-related gene. This gene demonstrated lower expression in COAD at the RNA level (Fig. 4A) and its expression level was significantly associated with survival time (*p* = 0.001; Table 1). Interestingly, we also observed that GDE1 expression in N-COAD was significantly lower than that in normal tissues (*p* < 1e−6) but higher than that in M-COAD (*p* = 0.003) (Fig. 4A). Furthermore, GDE1 was the most significant gene correlated with N-COAD subtype prognosis (Table 1).

**Table 1:** Top 10 significant prognosis in different histological type of COAD.

Gene	Gene expression direction	All COAD prognosis HR (*p*-value)	N-COAD prognosis HR (*p*-value)	M-COAD prognosis HR (*p*-value)
GDE1	Down^***^	0.41(0.001^**^	0.4(0.0017^**^	0.93(0.92)
DARS	Up^***^	2.1(0.0043^**^	2(0.0091^**^	2.9(0.1)
SLCO4A1	Up^***^	2(0.0066^**^	1.7(0.043^*^	3.1(0.087)
EPHB2	Up^***^	0.53(0.016^*^	0.51(0.019^*^	0.97(0.96)
B3GNT8	Down^***^	0.54(0.02^*^	0.53(0.022^*^	0.68(0.56)
DGUOK	Up^***^	1.7(0.029^*^	1.7(0.052)	2.3(0.2)
MARS2	Up^***^	0.57(0.03^*^	0.67(0.15)	1.1(0.92)
MAPKAPK5	Up^***^	0.57(0.031^*^	0.58(0.053)	0.35(0.099)
AURKA	Up^***^	0.59(0.041^*^	0.7(0.2)	1(0.98)
ALG1	Up^***^	0.6(0.054)	0.75(0.3)	0.57(0.36)

**Notes.**

HR represents the Hazard ratio.

Significances are cataloged as ^∗^*P* -value of 0.05–0.01, ^∗∗^*P*-value of 0.01 − 10^−6^, or ^∗∗∗^*P* < 10^−6^.

This table was ranked by the *p*-value of the prognosis of all COAD samples.

Although different histological subtypes of COAD showed similar prognosis profiles (Fig. 4B), we found that the correlation of GDE1 with prognosis was distinct in the N-COAD and M-COAD subtypes (Figs. 4C and 4D). Specifically, GDE1 was significantly associated with N-COAD prognosis (*p* = 0.0017), and N-COAD with high GDE1 expression demonstrated significantly longer survival time (Fig. 4C). In M-COAD, however, GDE1 was not correlated with prognosis (*p* = 0.92) (Fig. 4D). Taken together, our results showed that GDE1 was specially associated with N-COAD subtype prognosis, suggesting that it could be a potential novel cancer target for N-COAD.

## Discussion

Both the incidence and mortality of COAD have increased markedly in colorectal cancer in the past few decades (Guo et al., 2018). From a molecular basis, COAD results from multistep processes of aberration and accumulation, which drive malignant transformation of normal colon cells (Liu et al., 2017). However, the genetic changes responsible for the development and progression of COAD are still under investigation.

In this study, we obtained 2,114 metabolism-related DEGs in COAD, among which we identified GDE1 as the most significant gene related to prognosis and histological typing of COAD. This gene (also referred to as MIR16 or 363E6.2), localized on chromosome 16 (16p12.3) and containing six exons and five introns (https://www.ncbi.nlm.nih.gov), produces a 331-residue protein with an apparent molecular mass of 37.7 kDa (Bachmann, Duennebier & Mocz, 2006). The expression of GDE1 in mammalian tissues was first identified in 2000 (Zheng, Chen & Farquhar, 2000). It is an integral membrane glycoprotein and interacts with the RGS16 proteins, which regulates G protein signaling.

GDE1 also participates in glycerophosphoinositol-phosphodiesterase (GPI-PDE) activity. GDE1 possesses the evolutionarily conserved GDE domain. The GDE domain contains a putative catalytic motif (Zheng et al., 2003), which is important for GDE1-mediated hydrolysis of glycerophosphoinositol (GPI) (Zheng et al., 2003). GDE1 prefers GPI and some of its phosphorylated derivatives as potential substrates and demonstrates dramatic GPI-PDE activity (Zheng et al., 2003). As a GPI-PDE, GDE1 can further hydrolyze GPI to its final catabolic products, inositol and glycerol 3-phosphate (Zheng et al., 2003). Interestingly, inositol has been proven to show moderate anticancer activity in cell proliferation and differentiation (Bacić et al., 2010), and thus prevents the formation and incidence of several cancers alone or in combination with IP6, such as colon, breast, and lung cancer (Bacić et al., 2010; Vucenik & Shamsuddin, 2003; Bizzarri et al., 2016; Vucenik & Shamsuddin, 2006). Inositol is considered to be the parent compound of IP6 when all of its six carbons are attached to phosphate groups, and IP6 can be converted to inositol by removing all six phosphates (Vucenik & Shamsuddin, 2006). In addition, inositol is a precursor in the phosphatidylinositol cycle and conducts important second messengers in cellular signal transduction systems, such as phosphatidylinositol 4,5-bisphosphate (PIP2) and inositol 1,4,5-P3(IP3). Taken together, these studies show that inositol, as an enzymatic product of GDE1, plays a key role in anti-cancer.

Contra to the attention paid to the anti-cancer effects of inositol, little is known about the connection between GDE1 and cancer. In this study, we provide the first piece of evidence to show that GDE1 is an important tumor suppressor gene through the function of its metabolite inositol. Indeed, GDE1 demonstrated significantly lower expression in COAD tissues compared with normal tissues. Furthermore, its expression level showed a positive correlation with survival time; i.e., COAD patients with lower GDE1 expression displayed a shorter survival time. A possible explanation for this observation is that lower expression of GDE1 converted to less inositol, with less inositol resulting in poorer prognosis. Previous studies have indicated that increasing concentrations of inositol *in vivo* and *in vitro* may control cancer metastases and improve quality of life (Bacić et al., 2010; Vucenik & Shamsuddin, 2003; Vucenik & Shamsuddin, 2006; Lam et al., 2006; Abul Kalam, Shamsuddin & Ivana, 2005; Shamsuddin, Ullah & Chakravarthy, 1989; Carlomagno & Unfer, 2011; Fu et al., 2016; Nishino et al., 1999). Supporting evidence for this comes from our survival analysis results, in which higher expression of GDE1 showed better survival in COAD.

Furthermore, GDE1 showed different expression levels in different histological subtypes of COAD. Variant histological subtypes are reportedly associated with different survival outcomes (Hyngstrom et al., 2012) and treatments. For example, chemotherapy and radiotherapy have been administered to a higher percentage of patients with mucinous tumors than patients with non-mucinous adenocarcinomas (Hyngstrom et al., 2012). Individual therapy for different histological subtypes may result in an optimal effect. In our study, the expression of GDE1 in N-COAD was higher than that in M-COAD. In addition, N-COAD showed better survival with higher expression of GDE1. In contrast, there was no correlation between GDE1 and prognosis in M-COAD. Therefore, GDE1 may serve as a prognostic factor and candidate target in the N-COAD subtype.

## Conclusions

Our results suggest that GDE1 may be a cancer suppressor by up-regulating the inositol metabolism pathway. Its high association with N-COAD prognosis suggests this gene could also act as a promising cancer therapeutic target for this COAD subtype.

##  Supplemental Information

10.7717/peerj.8421/supp-1 Figure S1 Boxplot of log2 TPM between normal samples and tumor samples Green: the normal samples. Pink: the tumor samples.

10.7717/peerj.8421/supp-2 Figure S2 The correlation network of the up and downregulated genes in red module using cytoscape networking Magenta are the upregulated genes in red module. Blue are the down regulated genes in red module.

10.7717/peerj.8421/supp-3 Table S1 2144 DEGs in COAD 1224 highly expressed genes and 890 lowly expressed genes

10.7717/peerj.8421/supp-4 Table S2 199 genes in the red module

10.7717/peerj.8421/supp-5 Supplemental Information 1 Code of this article

## Additional Information and Declarations

### Data Availability

The following information was supplied regarding data availability: Raw data is available at The Cancer Genome Atlas (search term: COA): https://portal.gdc.cancer.gov/repository. The code is available as a Supplementary File.

## References

[ref-1] Abul Kalam M, Shamsuddin AM, Vucenik I (2005). IP6 & inositol in cancer prevention and therapy. Current Cancer Therapy Reviews.

[ref-2] Al-Tonbary Y, Darwish A, El-Hussein A (2013). Adenocarcinoma of the colon in children: case series and mini-review of the literature. Hematology/Oncology and Stem Cell Therapy.

[ref-3] Bachmann AS, Duennebier FF, Mocz G (2006). Genomic organization, characterization, and molecular 3D model of GDE1, a novel mammalian glycerophosphoinositol phosphodiesterase. Gene.

[ref-4] Bacić I, Druzijanić N, Karlo R, Skifić I, Jagić S (2010). Efficacy of IP6+inositol in the treatment of breast cancer patients receiving chemotherapy: prospective, randomized, pilot clinical study. Journal of Experimental & Clinical Cancer Research.

[ref-5] Bizzarri M, Dinicola S, Bevilacqua A, Cucina A (2016). Broad spectrum anticancer activity of myo-inositol and inositol hexakisphosphate. International Journal of Endocrinology.

[ref-6] Bray F, Ferlay J, Soerjomataram I, Siegel RL, Torre LA, Jemal A (2018). Global cancer statistics 2018: GLOBOCAN estimates of incidence and mortality worldwide for 36 cancers in 185 countries. CA: A Cancer Journal for Clinicians.

[ref-7] Brenner H, Kloor M, Pox CP (2014). Colorectal cancer. Lancet.

[ref-8] Brunk E, Sahoo S, Zielinski DC, Altunkaya A, Dräger A, Mih N, Gatto F, Nilsson A, Preciat Gonzalez GA, Aurich MK, Prlić A, Sastry A, Danielsdottir AD, Heinken A, Noronha A, Rose PW, Burley SK, Fleming RMT, Nielsen J, Thiele I, Palsson BO (2018). Recon3D enables a three-dimensional view of gene variation in human metabolism. Nature Biotechnology.

[ref-9] Carlomagno G, Unfer V (2011). Inositol safety: clinical evidences. European Review for Medical and Pharmacological Sciences.

[ref-10] Fu M, Song Y, Wen Z, Lu X, Cui L (2016). Inositol hexaphosphate and inositol inhibit colorectal cancer metastasis to the liver in BALB/c mice. Nutrients.

[ref-11] Guo T, Xie L, Zhao J, Song W, Dai W, Liu F, Zheng Y, Xu Y (2018). Trend analysis of morbidity and mortality of colorectal cancer in China from 1988 to 2009. Chinese Journal of Gastrointestinal Surgery.

[ref-12] Hanahan D, Weinberg RA (2011). Hallmarks of cancer: the next generation. Cell.

[ref-13] Hyngstrom JR, Hu CY, Xing Y, You YN, Feig BW, Skibber JM, Rodriguez-Bigas MA, Cormier JN, Chang GJ (2012). Clinicopathology and outcomes for mucinous and signet ring colorectal adenocarcinoma: analysis from the national cancer data base. Annals of Surgical Oncology.

[ref-14] Jacobsen A (2015). http://cran-r-project.org/package=cgdsr.

[ref-15] Ji M, Zhao X, Hu H, Jian F (2017). Primary colorectal cancer in 1092 cases: a five-year retrospective analysis. Academic Journal of Guangzhou Medical University.

[ref-16] Lam S, McWilliams A, LeRiche J, MacAulay C, Wattenberg L, Szabo E (2006). A phase I study of myo-inositol for lung cancer chemoprevention. Cancer Epidemiology, Biomarkers & Prevention.

[ref-17] Li Z, Zhang H (2016). Reprogramming of glucose, fatty acid and amino acid metabolism for cancer progression. Cellular and Molecular Life Science.

[ref-18] Liu H, Liu Z, Li K, Li S, Song L, Gong Z, Shi W, Yang H, Xu Y, Ning S, Ismail S, Chen Y (2017). TBL1XR1 predicts isolated tumor cells and micrometastasis in patients with TNM stage I/II colorectal cancer. Journal of Gastroenterology and Hepatology.

[ref-19] Liu S, Chen L, Xu Y (2018). Significance of PYK2 level as a prognosis predictor in patients with colon adenocarcinoma after surgical resection. OncoTargets and Therapy.

[ref-20] Ministry of Health of the People’s Republic of China (2016). China health and family planning statistical yearbook.

[ref-21] Nishino H, Murakoshi M, Masuda M, Tokuda H, Satomi Y, Onozuka M, Yamaguchi S, Bu P, Tsuruta A, Nosaka K, Baba M, Takasuka N (1999). Suppression of lung and liver carcinogenesis in mice by oral administration of myo-inositol. Anticancer Research.

[ref-22] Null RCTR, Team R, Null RCT (2009). R—a language and environment for statistical computing. Computing.

[ref-23] Roncucci L, Mariani F (2015). Prevention of colorectal cancer: how many tools do we have in our basket?. European Journal of Internal Medicine.

[ref-24] Shamsuddin AM, Ullah A, Chakravarthy AK (1989). Inositol and inositol hexaphosphate suppress cell proliferation and tumor formation in CD-1 mice. Carcinogenesis.

[ref-25] Thanikachalam K, Khan G (2019). Colorectal cancer and nutrition. Nutrients.

[ref-26] Vander Heiden MG, Cantley LC, Thompson CB (2009). Understanding the Warburg effect: the metabolic requirements of cell proliferation. Science.

[ref-27] Vucenik I, Shamsuddin AM (2003). Cancer inhibition by inositol hexaphosphate (IP6) and inositol: from laboratory to clinic. Journal of Nutrition.

[ref-28] Vucenik I, Shamsuddin AM (2006). Protection against cancer by dietary IP6 and inositol. Nutrition and Cancer.

[ref-29] World Cancer Research Fund/American Institute for Cancer Research (2007). Food, nutrition, physical activity, and the prevention of cancer: a global perspective.

[ref-30] Zheng B, Berrie CP, Corda D, Farquhar MG (2003). GDE1/MIR16 is a glycerophosphoinositol phosphodiesterase regulated by stimulation of G protein-coupled receptors. Proceedings of the National Academy of Sciences of the United States of America.

[ref-31] Zheng B, Chen D, Farquhar MG (2000). MIR16, a putative membrane glycerophosphodiester phosphodiesterase, interacts with RGS16. Proceedings of the National Academy of Sciences of the United States of America.

