# Supplementary figures and images for: Glycerophosphodiester phosphodiesterase 1 (GDE1) acts as a potential tumor suppressor and is a novel therapeutic target for non-mucin-producing colon adenocarcinoma
Source: PeerJ. 2020 Feb 11;8:e8421. doi: 10.7717/peerj.8421 (PMC7020812; doi:10.7717/peerj.8421)

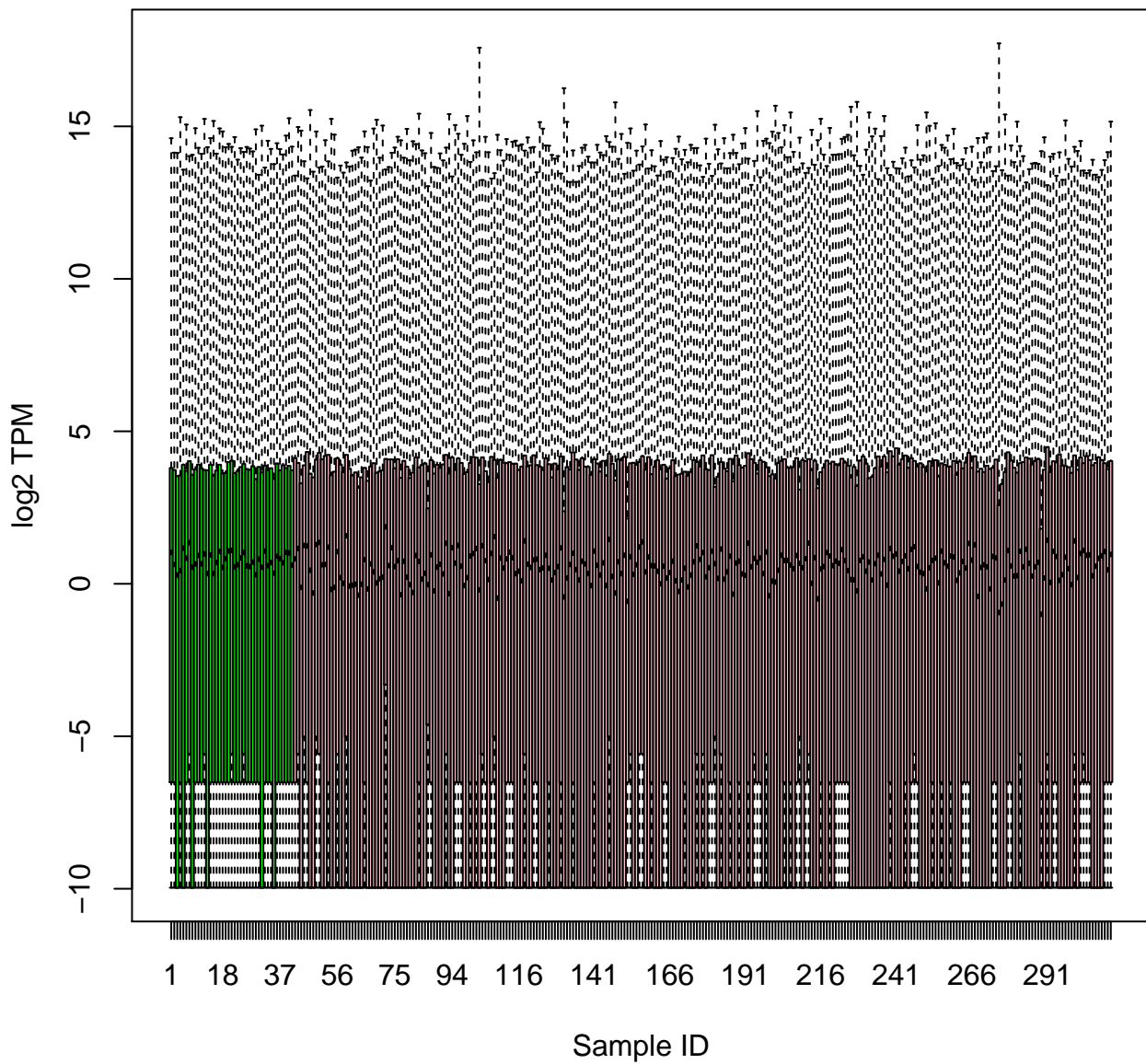

Supplement: Figure S1 — Green: the normal samples. Pink: the tumor samples. [file peerj-08-8421-s001.pdf]

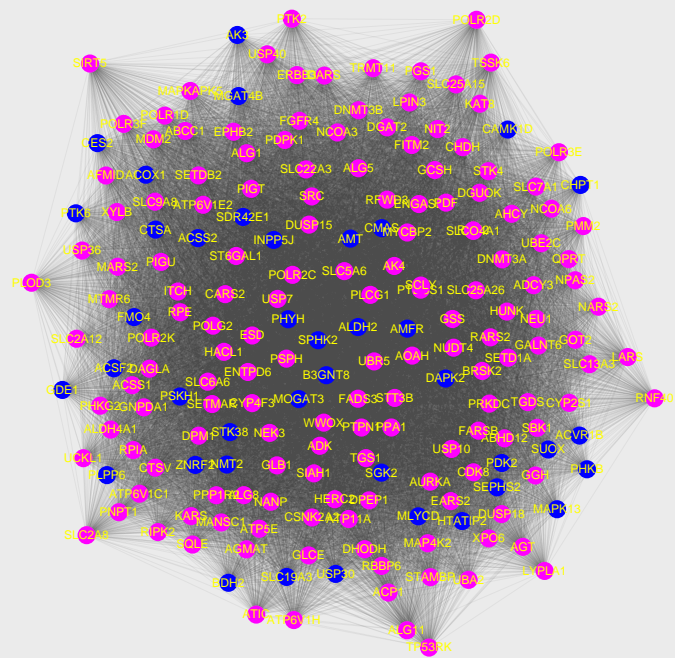

Supplement: Figure S2 — Magenta are the upregulated genes in red module. Blue are the down regulated genes in red module. [file peerj-08-8421-s002.pdf]
